# Monitoring Spontaneous Swallowing After Tracheostomy Using a Neck-Worn Electronic Stethoscope: A Pilot Study

**DOI:** 10.3390/jcm15082911

**Published:** 2026-04-11

**Authors:** Shin Matsumoto, Tetsuro Wada, Yukiyo Shimizu, Satoshi Fukuzawa, Yohei Teramoto, Haruna Nakazawa, Yasushi Hada, Kenji Suzuki, Keiji Tabuchi

**Affiliations:** 1 Department of Otolaryngology, Head and Neck Surgery, Institute of Medicine, University of Tsukuba, Tsukuba 305-8576, Japan; twada@md.tsukuba.ac.jp (T.W.); ktabuchi@md.tsukuba.ac.jp (K.T.); 2 Department of Rehabilitation Medicine, Institute of Medicine, University of Tsukuba, Tsukuba 305-8576, Japan; shimiyukig@md.tsukuba.ac.jp (Y.S.); y-hada@md.tsukuba.ac.jp (Y.H.); 3 Oral and Maxillofacial Surgery, Department of Clinical Medicine, Institute of Medicine, University of Tsukuba, Tsukuba 305-8576, Japan; fukuzawa.satoshi.gb@un.tsukuba.ac.jp; 4 Rehabilitation Department, University of Tsukuba Hospital, Tsukuba 305-8576, Japan; teramoto.yohei.kf@ms.hosp.tsukuba.ac.jp; 5 Nursing Department, Tsukuba University Hospital, Tsukuba 305-8576, Japan; nakazawa.haruna.kz@ms.hosp.tsukuba.ac.jp; 6 Institute of Systems and Information Engineering, University of Tsukuba, Tsukuba 305-8576, Japan; kenji@ieee.org

**Keywords:** tracheostomy, dysphagia, spontaneous swallowing frequency, neck-worn electronic stethoscope, reflexive pharyngeal swallow, artificial intelligence

## Abstract

**Background/Objectives**: Reduced spontaneous swallowing frequency (SSF) may reflect dysphagia. In this study, SSF was evaluated using a neck-worn electronic stethoscope (NWES), certified as a medical device in Japan, with artificial intelligence support in patients undergoing tracheostomy. **Methods**: This single-center observational study included tracheotomy patients who underwent swallowing assessment with an NWES between August 2024 and July 2025. Several variables were evaluated, including tracheostomy cannula cuff status and dietary status, assessed using the Functional Oral Intake Scale (FOIS). The Mann–Whitney U-test was applied, with SSF (/min) measured over 10 min using an NWES as the primary objective variable. Furthermore, Spearman’s correlation analysis was performed to examine the relationship between SSF (/min) and the pharyngeal saliva retention grade, which was determined using the Hyodo score during fiberoptic endoscopic evaluation of swallowing (FEES). **Results**: Eighteen patients who underwent tracheotomies were included in this study. SSF (/min) increased significantly when the tracheostomy cannula cuff was deflated (*p* = 0.049) and when the feeding status was FOIS ≥ 3 (*p* = 0.032) or FOIS ≥ 4 (*p* = 0.014). Spearman’s correlation analysis revealed a negative correlation between SSF (/min) and the pharyngeal saliva retention grade (ρ = −0.68, *p* = 0.0019). **Conclusions**: SSF measured with an NWES tended to increase with improved swallowing function, which is consistent with the outcomes of previous reports. The SSF measurement method used in this study may prove to be a useful clinical tool.

## 1. Introduction

Tracheostomy is a common procedure, with approximately 28,000 performed annually in Japan according to the 2022 National Database published by the Ministry of Health, Labor and Welfare [1]. Dysphagia is prevalent among patients undergoing tracheostomy, which can be attributed to the negative impact of the procedure on laryngeal closure [2] and swallowing reflexes [3]. While tracheostomy modifications, such as cuff deflation and speaking valve placement, may alter swallowing physiology, the evidence for this remains limited [4].

Different muscle activities have been reported to occur during cued and spontaneous swallowing [5]. Spontaneous swallowing is an airway defense reflex that prevents aspiration. While the spontaneous swallowing frequency (SSF) in healthy individuals is estimated to be 0.21–0.98/min [6], one study described a decrease in SSF in a specific post-tracheostomy patient population [7]. We hypothesized that patients who underwent tracheostomy would exhibit reduced SSF, reflecting dysphagia, and that SSF measurement would inform clinical decisions regarding tracheostomy management. However, uniform standards applicable across multiple facilities are required to test this hypothesis. Although SSF can be measured using methods such as electromyography and acoustic analysis, it has not yet been standardized.

Recently, a neck-worn electronic stethoscope (NWES) capable of recording swallowing sounds has been developed and approved as a medical device in Japan [8]. Such devices can be used in research to analyze swallowing function with the aid of artificial intelligence (AI), and the capability of this device to identify swallowing sounds has been validated in previous studies [9]. This study aimed to explore the clinical significance of SSF, determined using an NWES, in post-tracheostomy patients.

## 2. Materials and Methods

### 2.1. Study Design and Patients

This observational study was conducted between August 2024 and July 2025, targeting tracheostomized patients admitted to a single university hospital who required dysphagia assessment. The status of the tracheostomy cuff (inflated or deflated) was recorded, and dysphagia was assessed using an NWES in addition to routine examinations. To monitor swallowing movements, patients wore an NWES for approximately 10 min while not eating, and the number of swallows was measured. Measurements were performed without the use of a speaking valve. To ensure objectivity and minimize bias, all processes including data collection, entry, and analysis were performed by hospital personnel independently of the system developers. Patients with a history of neck reconstructive surgery, internal carotid artery stenosis, trauma, or burns to the neck were not treated using an NWES. Furthermore, patients with potentially obstructive lesions, such as uncontrolled esophageal or hypopharyngeal cancer, and patients who were unable to remain at rest or refused to wear the device were also excluded. After device placement, patients were evaluated for adverse events such as pain and skin disorders. By opting out, the Institutional Review Board exempted this observational study from the requirement of obtaining informed consent from the individual participants (approval number: R07-049).

### 2.2. Definitions of Explanatory Variables

Medical information, including patient age, sex, dietary intake, and tracheostomy cannula status, was obtained from chart review. Dietary status was assessed with the Functional Oral Intake Scale (FOIS) [10], which was determined using order information from electronic medical records to establish a solid endpoint. FOIS 3 was assigned when patients received both jelly food and tube feeding daily, and FOIS 4 was assigned when they received monophasic paste food. Pharyngeal saliva retention was graded on a 4-point scale by two experienced raters in accordance with the Hyodo dysphagia score [11,12] via fiberoptic endoscopic evaluation of swallowing (FEES). The grade of pharyngeal saliva retention based on the Hyodo score is described as follows: Grade 0, no retention; Grade 1, retention in the vallecula only; Grade 2, retention in the vallecula and piriform sinuses without laryngeal penetration; Grade 3, retention in the vallecula and piriform sinuses with laryngeal penetration.

### 2.3. Use of a Neck-Worn Electronic Stethoscope

**Figure 1:**
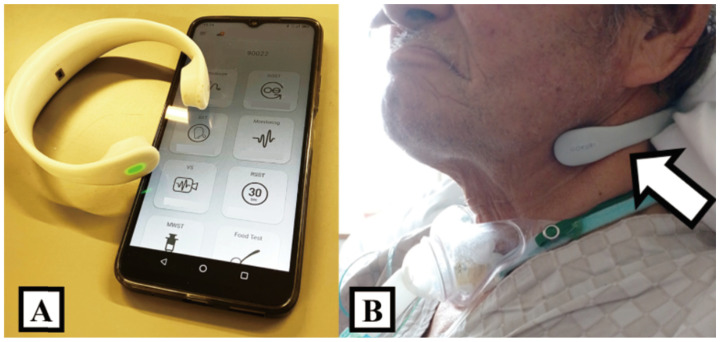
The figure displays the neck-worn electronic stethoscope used in this study. (**A**) Examination results are recorded on the cloud using a dedicated smart device. (**B**) The U-shaped device is worn at the position indicated by the arrow and clamped from behind the neck.

GOKURI^TM^ (PLIMES Inc., Tsukuba, Japan), an NWES, was utilized to evaluate SSF in this study. The NWES was placed so that its U-shaped tip was below the mandible (Figure 1A,B). This position, based on the advice of the developers, was slightly higher than the attachment site used during the development phase (C5 level of the cervical spinal cord) [13] and does not interfere with the tracheostomy cannula.

After the device was fitted, monitoring mode was initiated, and the patient was asked to swallow saliva once to confirm that the swallowing sound was recorded correctly. This time was excluded from the statistical analysis. The recorded swallowing frequency was divided by the measured time to calculate SSF (/min). If multiple measurements were performed for the same patient during hospitalization, the most recent test result was used.

### 2.4. Statistical Analysis

Data are presented as mean (standard deviation) for continuous variables and as numbers for categorical variables. The SSF (/min) for the study population is presented using histograms. Mann–Whitney U analysis was performed with SSF (/min) as the response variable and age, sex, cannula cuffed or cuff-deflated, and dietary status (FOIS ≥ 3 and FOIS ≥ 4) as the explanatory variables. Multivariate analysis was not performed due to the lack of cases. The significance level of the *p*-value was set at 0.05. To evaluate the predictive performance of SSF for cuff deflation, FOIS 3, and FOIS 4, receiver operating characteristic (ROC) curve analysis was performed, and areas under the curve (AUCs) were calculated. Spearman’s correlation analysis was used to evaluate the relationship between SSF and the mean pharyngeal saliva retention grade. Statistical analyses were performed using R version 4.4.1 (The R Foundation, Vienna, Austria).

## 3. Results

Overall, 25 tests were performed involving 18 patients. The study included thirteen men and five women, with a median age of 73 years (range 29–87 years). The indications for tracheotomy varied (Table 1).

**Figure 2:**
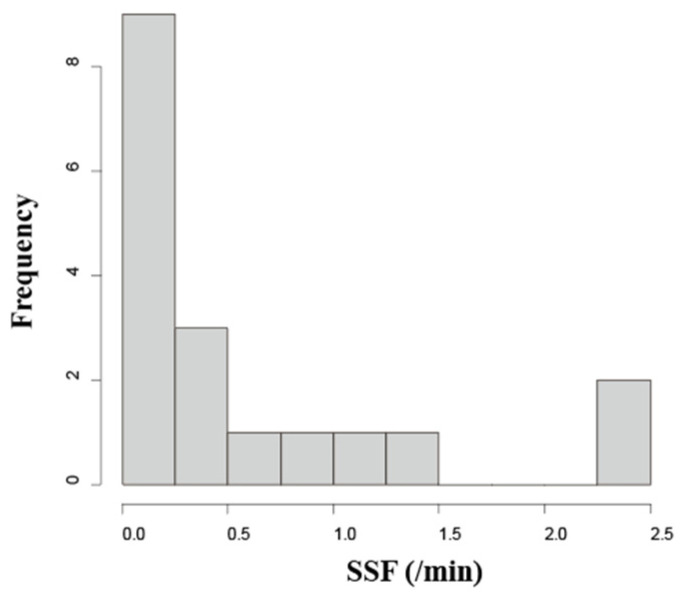
Histogram of spontaneous swallowing frequency in patients after tracheotomy.

The histograms of SSF (/min) indicated a non-normal distribution in the study population (Figure 2). In the univariate analysis, SSF (/min) significantly increased with tracheostomy tube cuff deflation (*p* = 0.049), FOIS ≥ 3 (*p* = 0.032), and FOIS ≥ 4 (*p* = 0.014) (Table 2). No adverse events, such as pain, skin disorders, or blood flow disorders, were observed during the 10 min of NWES.

**Figure 3:**
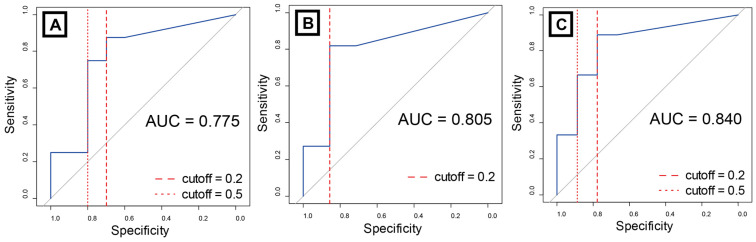
Receiver operating characteristics of spontaneous swallowing frequency. The areas under the curves for cuff deflation (**A**), FOIS 3 (**B**), and FOIS 4 (**C**) are 0.775, 0.805, and 0.840, respectively. The Youden Index cut-off point is 0.190 for all patients. The sensitivity and specificity of each method are listed in Table 3. Abbreviations: AUC, area under the curve; FOIS, Functional Oral Intake Scale.

In the ROC analysis, cuff deflation, FOIS 3, and FOIS 4 demonstrated good predictive performance, with AUCs of 0.775, 0.805, and 0.840, respectively (Figure 3). The Youden Index cut-off point was 0.190 across all three groups. When the SSF cutoff value was set at 0.2, the sensitivity and specificity were as follows: cuff deflation, 0.875 and 0.700; FOIS 3, 0.818 and 0.857; and FOIS 4, 0.889 and 0.778, respectively. When the cut-off value was set at 0.5, the specificity of each test increased to 0.800, 0.857, and 0.889, respectively (Table 3).

**Figure 4:**
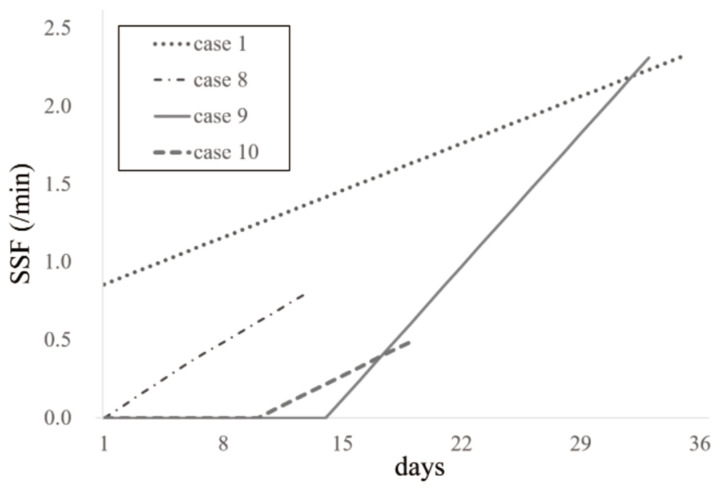
Time course of spontaneous swallowing frequency measured repeatedly.

The final SSF measurement was used in this study. For supplemental information, SSF values from cases measured multiple times are plotted (Figure 4). In all such cases, SSF increased over time.

**Figure 5:**
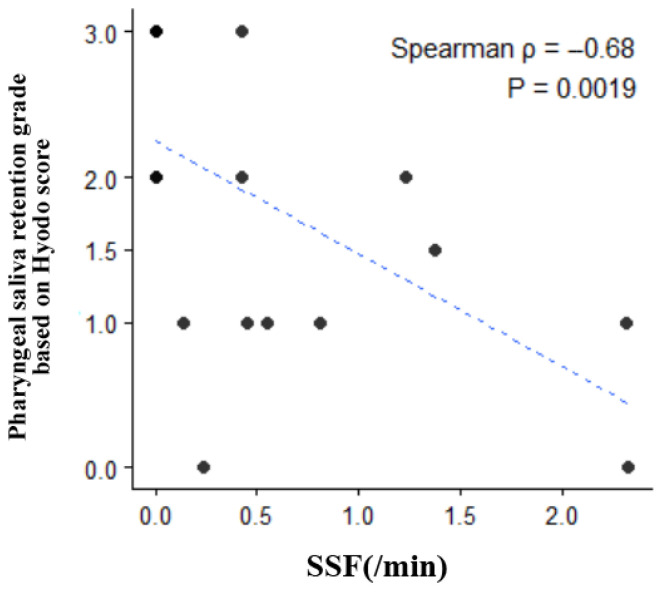
Spearman’s correlation between pharyngeal saliva retention grade and spontaneous swallowing frequency.

The grade of pharyngeal saliva retention, based on the Hyodo score as assessed through FEES, was almost consistent between the two raters (Figure 5). The grade of pharyngeal saliva retention showed a negative correlation with SSF (ρ = −0.68, *p* = 0.0019).

## 4. Discussion

In this study, we investigated SSF in post-tracheostomy patients using an NWES, certified as a medical device, with AI support. The results revealed that SSF tended to increase when the cannula was in a cuff-deflated state, or when FOIS ≥ 3, or ≥4. Furthermore, pharyngeal saliva retention on FEES tended to decrease as SSF increased. An increased SSF may reflect improved swallowing function. Due to its small sample size and subsequent limited statistical power, this study should be interpreted as an exploratory pilot study intended for hypothesis generation. To the best of our knowledge, this is the first report of SSF measurement using a certified medical device that can be standardized.

Salivary retention in the pharynx induces a spontaneous swallowing reflex, and it has been suggested that this physiological reflex prevents aspiration. Research has demonstrated that pharyngeal water stimulation induces reflexive pharyngeal swallowing and that this reflex is attenuated by anesthesia and smoking [14,15]. In the present study, the frequency of reflexive pharyngeal swallowing was assessed and recorded as the SSF. The reduction in pharyngeal saliva through the periodic spontaneous swallowing reflex may explain the association between a higher SSF and lower saliva retention grades in FEES. A literature review of SSFs was conducted to consider their utility as screening tools for identifying dysphagia [6]. The review identified a mean SSF of 0.98/min in a healthy young population and 0.21/min in a healthy older population. As various methods, including electromyography, acoustic/sound recording, respiratory bellows/transducers, and scoring by a trained observer, have been used to measure SSF, the lack of standardization remains a challenge. The NWES employed in the present study utilized AI to analyze the acquired acoustic data to determine swallowing activity. An NWES is easy to operate and, being certified as a medical device in Japan, enables multiple facilities to maintain a consistent environment. While NWESs are certified as medical devices and are available for use, their application for assessing swallowing function remains limited to research settings.

The application of NWESs has been extensively studied in recent years. Recent studies using an NWES have demonstrated that pharyngeal clearance time [16] and oral processing time [17] undergo age-related prolongation. Additionally, the impact of semi-solid food properties on swallowing behavior among older adults has been examined [18]. In the development of AI algorithms for NWESs, a convolutional neural network (CNN)-based deep learning model achieved a 98.1% accuracy in distinguishing swallowing sounds from other sounds with similar temporal characteristics [9]. This study applied the same AI algorithm that was previously validated.

The study population included patients who underwent a tracheostomy. In most cases presented in the histogram, the SSF was low compared to the previously reported mean values for healthy populations. Dysphagia commonly occurs after a tracheostomy, which weakens the glottic closure reflex [2], and an increased tracheostomy tube cuff pressure further impairs the swallowing reflex [3]. While reports on SSF in post-tracheostomy patients are limited, the mean SSF has been shown to increase after the decannulation of cuffed tubes in neurogenic dysphagia patients [7]. These reports suggest that post-tracheostomy patients, especially those with a cuffed tracheostomy tube, have impaired airway protective reflexes, supporting the observation that SSF is reduced.

In the present study, we explored specific applications of SSF, focusing on tracheostomy cannula management. For instance, we hypothesized that an improvement in SSF could be an indicator of tracheostomy tube cuff deflation, which is considered a necessary step for tracheostomy decannulation [19]. The benefits of early cuff deflation include a lower time to weaning, shorter ICU stay, and fewer complications [20]. One study on cuff deflation criteria introduced nine items, including medical stability, respiratory stability, and cuff secretion >1 mL per hour [21]. One important factor was the reduction in secretions above the cuff, which may reflect a recovery of the reflexive pharyngeal swallowing of secretions such as saliva. While the present study investigated the feasibility of using an elevated SSF as an objective indicator of cuff deflation, further studies are required to achieve this goal. In this study, cannula cuff deflation was performed at the discretion of the attending physician. Further exploratory studies are needed to determine the appropriate endpoints and cutoff values before any interventions, such as cuff deflation, can be performed based on the increased SSF. In the present study, cuff deflation and permission to consume FOIS 3 or 4 meals were considered candidate endpoints, and corresponding cutoff values were presented. Despite this, the potential applicability of SSF to tracheostomy cannula management was not directly validated in this study.

This small pilot case study is limited by its design, which does not allow for the exclusion of confounding factors. For instance, while factors such as cognitive function, history of pharyngeal disease, and disuse may potentially decrease SSF, the limited sample size of this study precluded the use of multivariate analysis. However, measuring SSF easily and safely with the support of AI is possible using an NWES, which has been certified as a medical device. It has been suggested that an increase in SSF reflects an improvement in the pharyngeal swallowing reflex. The SSF measurement method using an NWES is expected to be useful, as it can be standardized across multiple facilities. However, SSF is not yet at a stage where it can be utilized for clinical decision-making, and prospective interventional studies in larger cohorts are required before its integration into routine clinical practice.

## 5. Conclusions

SSF was measured using an NWES with the assistance of AI, and it was determined that an increase in SSF may reflect an improvement in swallowing function. However, this study is an exploratory pilot study focused on hypothesis generation, as the small sample size results in limited statistical power. Further research is warranted to utilize SSF in clinical practice.

## Figures and Tables

**Table 1:** Characteristics.

Case No	Age	Sex	Reasons for Tracheotomy and Dysphagia
1	<75	male	After surgery for a mediastinal abscess
2	<75	male	Previous treatment for oropharyngeal cancer, after treatment for severe pneumonia
3	≥75	male	Previous treatment for hypopharyngeal cancer, after treatment for cholecystitis
4	<75	female	Airway stenosis due to laryngeal cancer
5	<75	female	Hydrocephalus due to subarachnoid hemorrhage
6	<75	male	After treatment for acute epiglottitis
7	≥75	male	Cervical spine fixation following treatment for trauma
8	<75	female	After treatment for acute epiglottitis
9	<75	male	Cardiogenic shock
10	≥75	male	Cardiogenic shock
11	≥75	male	Recurrent laryngeal nerve paralysis due to thyroid cancer
12	≥75	male	Airway stenosis due to hypopharyngeal cancer
13	<75	female	Scoliosis due to malformation and aspiration pneumonia
14	<75	male	Epidural hematoma and epilepsy
15	<75	male	Airway stenosis due to laryngeal cancer
16	≥75	male	Airway stenosis due to laryngeal cancer
17	≥75	male	Previous treatment for laryngeal cancer, after treatment for severe pneumonia
18	≥75	female	Recurrent laryngeal nerve paralysis due to thyroid cancer

**Table 2:** Mann–Whitney U-test for spontaneous swallowing frequency.

			*n*	SSF		*p*
				Mean	sd	
age						0.437
	<75		10	0.77	0.93	
	≥75		8	0.32	0.42	
sex						0.685
	male		13	0.68	0.86	
	female		5	0.30	0.34	
cuff deflation						**0.049**
	no		10	0.32	0.54	
	yes		8	0.89	0.91	
FOIS ≥ 3						**0.032**
	no		7	0.20	0.46	
	yes		11	0.81	0.84	
FOIS ≥ 4						**0.014**
	no		9	0.20	0.41	
	yes		9	0.94	0.87	

Bold text indicates a statistically significant difference at a *p*-value < 0.05. Abbreviations: SSF, spontaneous swallowing frequency; FOIS, Functional Oral Intake Scale.

**Table 3:** Sensitivity and specificity corresponding to the cutoff values of spontaneous swallowing frequency.

Cutoff Value	Cuff Deflation (AUC = 0.775)		FOIS ≥ 3 (AUC = 0.805)		FOIS ≥ 4 (AUC = 0.840)	
	Specificity	Sensitivity	Specificity	Sensitivity	Specificity	Sensitivity
0.1	0.600	0.875	0.714	0.818	0.667	0.889
0.2	0.700	0.875	0.857	0.818	0.778	0.889
0.3	0.700	0.750	0.857	0.727	0.778	0.778
0.4	0.700	0.750	0.857	0.727	0.778	0.778
0.5	0.800	0.500	0.857	0.455	0.889	0.556

AUC, area under the curve; FOIS, Functional Oral Intake Scale.

## Data Availability

The datasets generated, analyzed, or both during this study are not publicly available because of the protection of personal information but can be obtained from the corresponding author upon reasonable request.

## Abbreviations

The following abbreviations are used in this manuscript:

SSF	Spontaneous swallowing frequency
NWES	Neck-worn electronic stethoscope
FOIS	Functional Oral Intake Scale
FEES	Fiberoptic endoscopic evaluation of swallowing
AI	Artificial intelligence
ROC	Receiver operating characteristic
AUC	Area under the curve
CNN	Convolutional neural network
ICU	Intensive care unit
sd	Standard deviation
